# A framework for the assessment and implementation of diagnostics in outbreak situations

**DOI:** 10.4102/ajlm.v5i3.494

**Published:** 2016-10-31

**Authors:** Elliot P. Cowan

**Affiliations:** 1 Partners in Diagnostics, LLC, Rockville, Maryland, United States

## Abstract

**Observation:**

Outbreak situations require in vitro diagnostics (IVDs) to identify those who are infected and to track the infectious agent in the population. However, such IVDs are typically not available and must be developed. In addition, the process of IVD development, assessment, and implementation are very time and resource intensive. Recognising the extraordinary public health need for IVDs in an outbreak situation, streamlined processes are needed to provide tests that meet the standard of a reasonable assurance of safety and effectiveness in the shortest amount of time. These IVDs are designated for outbreak use.

**Addressing Issues:**

This paper presents a pathway to the outbreak use of IVDs that can be considered by countries experiencing an outbreak situation. It takes into account recognition of the outbreak, product development, regulatory evaluation, implementation, and monitoring of the outbreak-use test. Streamlined assessment programmes for emergency-use tests have been established by the US Food and Drug Administration and the World Health Organization. These programmes take into account test requirements for the country in which the outbreak exists. Therefore, countries can consider adopting these tests without the need to conduct expensive and time consuming assessments, such as performance studies. Key responsible parties are identified for each step of the pathway, recognising that transparency and communication among all parties are critical.

## Introduction

Medical devices used for in vitro diagnostics (IVDs) are at the frontline of medical decision-making, which ranges from individual patient management to addressing public health crises. This central role for IVDs means that they must be safe, effective and reliable. Regulatory oversight of IVDs provides assurance by assessing both the performance claims made by manufacturer and the systems they have in place in their facilities to maintain product quality. However, all IVDs are not regulated in the same way. Typically, the higher the risk associated with an IVD, the more detailed the assessment.

This approach should be flexible in an outbreak situation. Outbreaks are associated with infectious agents that pose a grave danger to the population, and therefore the risk associated with an IVD that will be used to assist in the diagnosis of affected individuals is high. However, there is also an urgent need to make the IVD available as quickly as possible. Time is of the essence to limit the spread of the infectious agent and its associated morbidity and mortality. In this case, it is not possible for the assessment stringency to be consistent with the risk. There is an urgent need, then, to dramatically shorten the review process while ensuring the benefits of the IVD outweigh its risks. This paper describes a pathway for the evaluation and regulation of IVDs essential to respond to an infectious disease outbreak when no adequate, approved diagnostic tests for that infectious agent are currently available.

## A pathway to outbreak use in vitro diagnostics

An outbreak-use IVD is an IVD that is restricted to use during an outbreak situation to meet the critical public health need posed by the outbreak. It is a test that has been determined to have a reasonable assurance of safety and effectiveness in the shortest possible time. ‘Reasonable assurance’ means the information provided for assessment will be: (1) limited only to that necessary to demonstrate that the IVD is capable of fulfilling the goal of correctly identifying as many infected individuals as the product technology is capable of; and (2) sufficient to show that the benefits of using the product outweigh its risks. ‘Shortest possible time’ means identifying and overcoming test development and performance assessment barriers and using a process that has a clear, direct path, recognising the extraordinary threat to public health posed by the outbreak.

**FIGURE 1:**
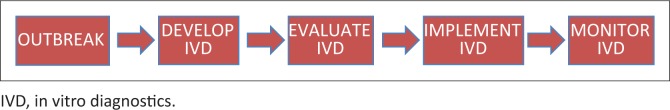
Pathway toward implementation of outbreak-use in vitro diagnostics.

Outbreak-use IVDs should be restricted to use during the outbreak. When the outbreak has officially ended, outbreak-use IVDs should be neither distributed, nor procured, nor used for clinical purposes. Rather, the manufacturer is encouraged to seek a general clinical use claim for its test. However, valuable information on test performance can be obtained during outbreaks to support a conventional regulatory assessment. Figure 1 shows the overall approach for implementing IVDs for outbreak use.

Each of these steps will be examined in the context of the development of tests that have a reasonable assurance of safety and effectiveness. This pathway is not specific to a particular infectious agent, but rather is a general framework intended to be applicable to any outbreak situation for which there is not a previously-accepted IVD.

## Outbreak recognition

An outbreak situation must be recognised and the infectious agent that is responsible for the outbreak identified to enable a cascade of actions that will facilitate both the development of tests and the regulatory assessment of those tests. This is a starting point for the process of IVD development, involving several parties.

The United States Department of Health and Human Services, through the Food and Drug Administration (FDA), and the World Health Organization (WHO), each has a mechanism to evaluate/authorise products for use in emergency situations and these authorised products can play a critical role in an outbreak (see below). However, the FDA, with input from either the US Centers for Disease Control and Prevention (CDC) or other US government agencies, and WHO must each officially recognise that a public health outbreak situation exists in order to invoke their respective review and performance evaluation mechanisms. Therefore, public health authorities in the country experiencing the outbreak must make the CDC (or other US agencies) and the WHO aware of the outbreak to enable them to recognise the outbreak situation as soon as possible to enable their respective streamlined emergency test assessment procedures to be officially declared.

Manufacturers must receive from global public health authorities, such as the CDC and WHO, accurate information on the nature of the outbreak infectious agent, its biology and clinical manifestations. This is necessary to identify target(s) that are most appropriate for detection in infected individuals. Regulatory authorities require this information to assess safety and effectiveness. Therefore, consistent, accurate and detailed information about the infectious agent must be widely disseminated.

In addition, manufacturers require clinical specimens to aid in test development and validate test performance. Regulatory authorities require these specimens to aid them in their assessment and post-market activities. Therefore, well-characterised clinical specimens must be made available and accessible from endemic areas through coordination by public health agencies working together with Ministries of Health.

At the end of this phase:

- There should be a conclusive identification of the specific infectious agent, with as many accompanying details as possible. This should include the virulence of the organism and the potential danger to individuals obtaining and testing specimens.
- All appropriate stakeholders, including IVD manufacturers, will be informed of the outbreak and all available information on the infectious agent.
- The FDA and WHO will recognise that an outbreak situation exists, to enable emergency authorisations for tests.
- The most appropriate diagnostic targets, intended use population(s), testing setting(s) and specimen type(s) will be identified.
- Clinical specimens will be available to test manufacturers for test development, and there will be no administrative barriers to obtaining those specimens.

## Development of in vitro diagnostics

Next, candidate IVDs for the outbreak infectious agent are developed. However, this process takes time and cannot be expected to meet the immediate need. Therefore, initial tests developed and deployed by public health agencies, such as the CDC, for research and surveillance purposes, once rapidly demonstrated to have diagnostic performance capabilities, will play a critical role in identifying and diagnosing infected individuals at the beginning of the outbreak.

This will allow commercial test manufacturers the time needed to rapidly develop new tests to meet the outbreak need or modify existing test platforms to detect the outbreak infection and meet the needs of a potential surge in newly-infected individuals. Existing test platforms may or may not have undergone a regulatory assessment. A target product profile must be developed that identifies the specific characteristics required for the outbreak use IVD and communicate it to potential manufacturers. Potential test developers/manufacturers should be identified that are most likely to develop and produce an outbreak use IVD in the shortest possible time, preferably to have the capability to consistently produce a quality test at a high volume and have adequate distribution channels in the endemic areas. They should be provided with incentives to support an acceptable business case for taking on test development. These may include funding to offset the cost of well-characterised specimen procurement and in-field studies, if required, which are often the major costs in bringing diagnostic products to market.

There are a number of additional challenges that must be addressed at this step, including technical barriers (for example, it is much easier to develop a nucleic acid test than a serological test); the time required to develop a test that is adequate to meet the needs of the outbreak situation; access to specimens from endemic areas for test development; the ability to manufacture the test at a scale sufficient to meet the need; and identifying manufacturers willing to develop and produce tests that will likely only be used during an outbreak situation. When successful, manufacturers will be prepared to submit their outbreak-use IVDs for emergency regulatory assessment.

## Evaluation of in vitro diagnostics

All IVDs for outbreak use should be assessed for safety and effectiveness to determine that the benefits of using the IVD outweigh the risks, and to provide some assurance that the IVD is manufactured under an appropriate quality system to assure that the products are produced, stored, and distributed in compliance with current good manufacturing practices. This is necessary to reasonably assure two equally important and essential elements: the test is capable of detecting the outbreak infectious agent; and it will consistently perform as expected. This is done through the review of scientific and surveillance information submitted by the test developer both before and after the regulatory evaluation.

We can consider three routes to acceptance of an IVD for outbreak use:

- Assessment of an IVD that has received an FDA Emergency Use Authorisation [EUA], a mechanism that assesses medical products for use in designated public health emergency situations, including IVDs for use in outbreaks.^1^
- Assessment of an IVD that has been found acceptable through the WHO Emergency Use Assessment and Listing (EUAL) mechanism, which also assesses medical products for use in designated public health emergency situations, including IVDs for use in outbreaks.^2^
- Country assessment of an IVD that has not been previously assessed.

**FIGURE 2:**
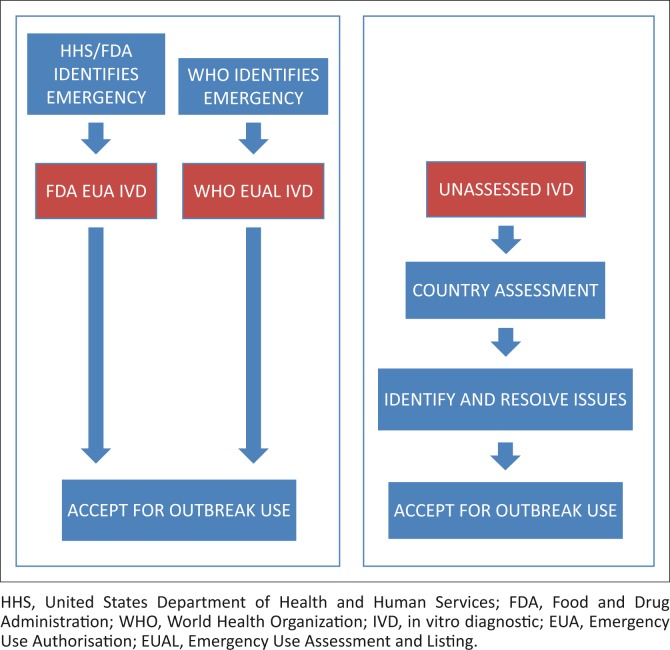
Assessment scheme for adoption of outbreak-use in vitro diagnostics.

Figure 2 is an overview of these routes and the overall recommended steps a country may follow for acceptance of an outbreak-use IVD for each of the pathways.

## Emergency Use Authorisation and Emergency Use Assessment and Listing

As discussed above, the FDA and/or WHO must recognise an outbreak situation in order to use its respective emergency assessment declaration mechanism for IVDs. When that happens, the FDA EUA and WHO EUAL pathways are each designed to provide reasonable assurance of safety and performance for an IVD for outbreak diagnostic use (and limited to such use) in the regions where the outbreak has occurred. Both the EUA and the EUAL take into account such factors as outbreak agent diversity, the ability of the product to function (ease of use) and its stability in the outbreak area environment, and potential interfering conditions, among others, consistent with the target product profile. Therefore, tests that have been successfully evaluated by the EUA or EUAL processes should be considered appropriate for outbreak use without further assessment. A country should, however, obtain from the test manufacturer:

- Official documentation from the FDA or WHO to verify EUA or EUAL status.
- A signed statement to attest that the outbreak use product provided to the country is identical to that evaluated for EUA or EUAL (the same manufacturing site, the same product version, and the same manufacturing procedures).

Tests could then be accepted for outbreak use immediately upon verification of EUA or EUAL status. However, it is important to note that the EUA process does not assess stability of the IVD at appropriate conditions of temperature, humidity, dust, altitude and ruggedness for a particular country other than the United States. Countries that have the ability and resources should conduct these studies for an EUA product. Countries that do not would be best served by implementing tests that have EUAL status, since the WHO process includes this necessary stability evaluation.

## Country assessment previously unassessed in vitro diagnostics

IVD manufacturers often seek acceptance for outbreak use directly from the country experiencing the outbreak without having EUA or EUAL status for its test. In this case, countries should assess the candidate tests according to the level of stringency used by the EUA and EUAL processes, which rely heavily on information submitted by the manufacturer. The country may choose to conduct its own performance assessment; however, these studies should be very limited and designed only to confirm that the test performs as expected. Studies done by the country should *not* be intended to establish test performance. These studies consume valuable time and are unnecessary at this stage. Rather, the focus should be on a limited verification of key manufacturer claims, including appropriate stability studies, as discussed above. It should be noted that countries affected by outbreaks often have limited capacity to assess IVDs on their own and require the assistance of public health agencies to do so.

When faced with an outbreak situation, countries should consider that both the FDA and the WHO have established fast-track ‘emergency use’ review pathways to evaluate the known and potential benefits of tests, both commercial and non-commercial (such as those tests developed by the CDC), which can be used to diagnose emerging, life-threatening, infectious diseases when no available FDA-approved/cleared test or WHO-prequalified test is available to healthcare providers. The authorisation for these tests is based on the totality of available scientific evidence presented to the FDA or the WHO by the test developer and is available on-line to public health authorities assessing which test is the most suitable for use in their country.^1,2^ Tests that have not been reviewed by a recognised regulatory body may certainly be assessed in-country, but it should be recognised that appropriate, well-designed studies may be costly and can delay the implementation of the IVD when it is critically needed.

## Implementation

While much of the responsibility for implementation lies with procurement bodies and agencies responsible for maintaining the supply chain, there are a number of actions affecting implementation that are related to the regulation of the outbreak-use IVD. Communication of what tests are acceptable for outbreak use is necessary to ensure that only tests that have met acceptable criteria are used in the outbreak. For example, the FDA establishes ‘conditions of authorisation’ for every EUA test it evaluates. This may refer to who may distribute the test, who may use the test and which individuals should be tested. Users must be trained in the operation of the test, including specimen preparation, running the test, and interpreting the test result. Training must also include clearly communicating the limitations and capabilities of the test to ensure that test operators use the test properly and only with allowable specimens. Successful implementation will see a distribution system that supplies adequate numbers of tests to areas affected by the outbreak, and users will be qualified to conduct and interpret the test.

## Monitoring

Given the critical role of the IVD in the outbreak and the limited studies that supported its acceptance, the IVD should be monitored for performance after it is introduced into clinical use. Monitoring can be addressed in a number of ways. Each country must decide which of these options is appropriate to monitor tests in clinical use during the outbreak situation.

### Adverse event reporting

There is an expectation that the manufacturer will track adverse events (including any product quality issues, especially those that lead to incorrect test results) associated with its test. This is required for both EUA and EUAL products, with reporting of events to the FDA and WHO, respectively, and should be a requirement by countries assessing tests for outbreak use. To support this, while each country should generate a reporting system for adverse events and a monitoring plan for the outbreak use IVDs it implements, there should also be a regional adverse event reporting system that coordinates responses to issues that arise quickly and effectively.

However, adverse event reporting systems cannot work unless test users are trained to understand what constitutes an adverse event and how to identify and report product-related issues. There must also be a culture that encourages test users to report adverse events and any product-related issues, and the test labeling must have clear instructions on what to do in the case of an adverse event.

### Periodic panel testing

Users periodically test panels of well-characterised specimens. This will assess the proficiency of test users and monitor ongoing test performance.

### Lot testing

Newly-received test kit lots are tested using a panel of specimens as a condition for acceptance into the country for outbreak use.

### Surveillance testing

Clinical specimens are periodically sent to a reference laboratory for testing to determine agreement with field test results (both specimens positive for the outbreak agent and specimens negative for the outbreak agent) to monitor for false positive and false negative test results. This is typically done from predetermined sites designated as surveillance sites.

### Accumulation of performance data to support World Health Organization prequalification

As a condition of EUAL, a manufacturer commits to filing for prequalification for its test. This means that the manufacturer will gather test performance data while the test is being used in the outbreak situation. FDA similarly encourages developers to work towards gathering EUA test performance data to obtain permanent status of clearance or approval for their test as, after the epidemic is declared over, the EUA version of a test is no longer allowed to be on the market.

Monitoring should be implemented as soon as possible after introduction of the IVD into clinical use and continue throughout its use during the outbreak.

## Conclusion

Each portion of the pathway that ultimately leads to the introduction of an outbreak-use IVD requires the involvement and cooperation of multiple bodies, each with critical roles and responsibilities. This includes public health agencies, Ministries of Health, regional regulatory bodies (such as the Pan-African Harmonisation Working Party^3^), the test user, and the test manufacturer. Table 1 shows proposed roles and responsibilities. Note that nearly every party is involved in some way in each of the steps of the outbreak-use pathway. This means that, whereas each participant in the pathway, by virtue of its expertise, has a particular and essential role in bringing forward and maintaining the quality of outbreak use IVDs, success depends upon all of the participants working together. The foundation of this cooperative approach is transparency and communication.

**TABLE 1:** Summary of roles and responsibilities for outbreak-use in vitro diagnostics

Responsible party	Roles and responsibilities
Appropriate public health agency (e.g., WHO, local WHO offices, CDC)	Recognise and communicate outbreak situation and epidemiology. Assist with identification of outbreak agent. Develop and deploy initial tests for outbreak agent. Assist Ministries of Health in disseminating information on the outbreak. Develop a TPP for the outbreak use IVD, and communicate to test developers/manufacturers. Identify test developers/manufacturers and development of incentives by philanthropic organisations. Connect manufacturers with sources of specimens for test development. Post information on the acceptance of outbreak use IVDs on its websites. Implement and maintain a laboratory listserv to facilitate communication within the laboratory community. Assist in monitoring activities.
Ministry of Health	Maintain a list of outbreak-use IVDs that are acceptable for use. Disseminate information on the outbreak, in association with public health agencies. Streamline administrative/legal issues to make specimens available. Ensure policies are in place to accept EUA or EUAL products for outbreak use. Oversee evaluation of products for which manufacturers seek outbreak use acceptance directly from the outbreak country (provide experts, ensure appropriate assessment). Notify agencies responsible for procurement when an IVD is accepted for outbreak use. Notify agencies responsible for procurement and public health bodies when an IVD is accepted for outbreak use. Ensure that users are trained to recognise issues and adverse events. Create and support professional environment that empowers and encourages users to report issues. Facilitate periodic panel testing as part of a monitoring program for outbreak-use IVDs, as appropriate. Oversee lot release testing, as appropriate. Coordinate surveillance testing, as appropriate.
Regional regulatory body	Assist with the assessment of potential outbreak-use IVDs. Assist with outbreak-use IVD monitoring, as needed.
Test user	Use tests consistent with their intended use. Report adverse events and product-related issues. Participate in monitoring activities, as appropriate.
Test manufacturer	Develop outbreak-use IVDs. Communicate with public health bodies and Ministries of Health on tests’ development progress, as appropriate. Conduct training of the intended users of the test after acceptance for outbreak use. Maintain quality of the product. Track adverse events and address product-related issues by identifying the root cause of problems and implementing corrective and preventive actions consistent with quality management system requirements.

WHO, World Health Organization; CDC, US Centers for Disease Control and Prevention; TPP, target product profile; IVD, in vitro diagnostic; EUA, Emergency Use Authorisation; EUAL, Emergency Use Assessment and Listing.

A transparent process is one in which information is freely available to as many people as possible. In the context of an outbreak situation, this includes the identification of the outbreak agent, the clinical course of the disease, and availability of potential targets around which a test could be developed (i.e., nucleic acid sequences, antigens, etc.), variants of the agent, epidemiology (location, movement, etc.), well-characterised specimens for test development and monitoring of test performance once the outbreak-use IVD is in clinical use, and simple and clear policies and procedures to accept a test for outbreak use.

Communication is the tool for transparency, and is necessary at all levels. This includes communication among those identifying the outbreak source, between those identifying the outbreak source and the product developers, the product developers and the regulators, public health authorities and clinicians, and all involved parties and the public. Of course, there are limits to transparency and communication. For example, a test manufacturer should not be expected to disclose proprietary information for its test. In general, though, a culture of information and specimen sharing by the countries most affected, and eventually the availability panels of well-characterised specimens for assay-to-assay comparison studies, are critical to the development, performance validation, performance review, and implementation of outbreak-use IVDs.

BOX 1: Lessons Learned.

**Lessons Learned:**

- Given the critical need and role for diagnostics in an outbreak situation, affected countries must have an efficient pathway toward development and implementation of tests for outbreak use.
- Public health agencies that may already have developed tests for research use and surveillance purposes, such as the US CDC, must be engaged as early as possible to begin the process of validating their tests for use in diagnosing and identifying infected individuals.
- Emergency assessment programs at FDA and WHO offer countries the opportunity to streamline the acceptance and implementation of outbreak use IVDs.
- The pathway to an outbreak use IVD requires transparency and communication among all responsible parties and with the public.
